# Atypical hyperpachymorph *Trypanosoma (Nannomonas) congolense* forest-type in a dog returning from Senegal

**DOI:** 10.1051/parasite/2012193239

**Published:** 2012-08-15

**Authors:** M. Desquesnes, S. Ravel, J.-Y. Deschamps, B. Polack, F. Roux

**Affiliations:** 1 Centre de Coopération Internationale en Recherche Agronomique pour le Développement (CIRAD), UMR Intertryp, 34398 Montpellier, France. Faculty of Veterinary Medicine, Kasetsart University Chatuchak, Bangkok 10900 Thailand; 2 Institut de Recherche pour le Développement (IRD), UMR Intertryp, LRCT Campus International de Baillarguet 34000 Montpellier France; 3 Emergency and Critical Care Unit, LUNAM University, ONIRIS, Nantes-Atlantic College of Veterinary Medicine, Food Science and Engineering La Chantrerie BP 40706 44307 Nantes France; 4 Université Paris-Est, École Nationale Vétérinaire d’Alfort, Unité de Parasitologie 94704 Maisons-Alfort France; 5 Université Paris-Est, École Nationale Vétérinaire d’Alfort, Unité d’Urgences et Soins Intensifs 94704 Maisons-Alfort France

**Keywords:** *Trypanosoma (Nannomonas) congolense* forest-type, dog, *montgomeryi*-form, hyperpachymorph, PCR, Giemsa-smear, *Trypanosoma (Nannomonas) congolense* type forêt, chien, forme *montgomeryi*, hyperpachymorphe, PCR, frottis Giemsa

## Abstract

*Trypanosoma congolense* forest-type was identified by PCR in France, in a dog returning from Senegal. This paper describes the morphological features of the parasite on Giemsa-stained smears. Slender forms and “latent bodies” represent 30.4% and 20.4%, respectively. Some rosettes have been observed (0.8%). The predominant form (48.4%) is stumpy, close to “*montgomeryi*form”, but it is unusually broad, with a width/length ratio (WLr) of 0.40-0.55, while that of “*montgomeryi*-forms” is close to 0.3. To the best of our knowledge, this is the first description of such a form of *T. (Nannomonas)*. Also unusual, the shape of the cytoplasm appears to be tightened by an “S-” or “C-” shaped flagellum. We propose naming this peculiar morphotype “hyperpachymorph”, and adding its description to that of *T. congolense* forest-type. Thus *T. (Nannomonas)* forms would include: sphaeromorph or “latent bodyform” (globular), hyperleptomorph (*rodhaini*-form, very long and slender, with a free flagellum); leptomorph (*simiae*-form, slender, with a free flagellum); isomorph (*congolense*-form, short, generally without a free flagellum); pachymorph (*montgomeryi*-form, short and stout; 0.25 < WLr < 0.34, without a free flagellum), and hyperpachymorph (“hyper-*montgomeryi*-form”, short and very stout; 0.35 < WLr < 0.7, without a free flagellum).

## Abstract (fr)

*Trypanosoma congolense* type forêt a été identifié par PCR en France, chez un chien de retour du Sénégal. Cette publication décrit les divers aspects morphologiques du parasite sur des frottis sanguins colorés au Giemsa. Les formes fines (*slender*) et les corps latents représentent respectivement 30,4 % et 20,4 % des parasites. Quelques rosettes ont été observées (0,8 %). Le morphotype prédominant (48,4 %) est trapu (*stumpy*), proches des formes “*montgomeryi*”, mais d’une largeur inhabituelle, avec un rapport largeur/longueur (rl/L) de 0,40-0,55, alors que celui des formes *montgomeryi* précédemment décrites est de l’ordre de 0,3. À notre connaissance, ceci est la première description de tels morphotypes de *T. (Nannomonas)*. La forme du cytoplasme qui semble “tendu” par un flagelle en forme de “S” ou de “C” est également surprenante. Nous proposons de nommer ce morphotype “hyperpachymorphe”, et de l’ajouter à la description de *T. congolense* type forêt. Ainsi, le sous-genre *T. (Nannomonas)* présenterait six morphotypes (du plus fin au plus large) : hyperleptomorphe (forme *rodhaini*, très long et mince avec flagelle libre); leptomorphe (forme *simiae*, mince avec flagelle libre), isomorphe (forme *congolense*, court généralement, sans flagelle libre), pachymorphe (forme *montgomeryi*, court et large (0,25 < rl/L < 0,34), sans flagelle libre), hyperpachymorphe (forme “hyper-*montgomeryi*”, court et très trapu (0,35 < rl/L < 0,7), au cytoplasme clair et sans flagelle libre) et sphaeromorphe ou “corps latent” (globulaire).

## Introduction

Identification of trypanosomes in mammals has long been based on the morphology and morphometry of the parasite in Giemsa-stained preparations in the host and vector, in conjunction with host and vector ranges, including xenodiagnosis (Hoare, 1972). Molecular biology brought about significant improvements in species identification, with the development of DNA probes and PCR assays (Dickin & Gibson, 1989; Moser *et al.*, 1989; Masiga *et al.*, 1992). Combined with parasitological and epidemiological data, molecular characteristics revealed by PCR or sequencing, may lead to accurate identification, and even to the construction of phylogenetic trees. These various tools may be used for the definition of species, particularly problematic in the case of protozoan parasites that do not include differentiated gametes in their cycle, such as trypanosomes which often proliferate more by asexual than sexual reproduction (Gibson, 2007). PCR, the most convenient molecular technique for trypanosome detection and identification, can point to various taxonomic levels (Desquesnes & Dávila, 2002). In the sub-genus *T. (Nannomonas)*, five-six taxa have been described (Gibson, 2007).

In the present paper, subsequent to the molecular confirmation of an infection with a single taxon of *Trypanosoma congolense* forest-type (Masiga *et al.*, 1992) in a dog returning from a stay in Senegal (molecular identification and clinical history are presented elsewhere), we describe the parasite morphology, with a special emphasis on a peculiar form observed on Giemsa-stained blood smears, never before described. We propose adding this novel form to the described forms of the taxon *T. (Nannomonas) congolense* forest-type.

## Material and Methods

During a stay in Senegal, Cap Skirring, Casamança, which began on November 6th, 2010, a five-year-old male Shih Tzu dog became lethargic, weak in the hindquarters and inappetant, although afebrile, on November 29th, 23 days after his arrival. By December 4th, his condition had deteriorated and he began to vocalize. For this reason, his owner decided to fly back to France on the evening of December fourth. The animal presented on December 5th at the Emergency and Critical Care Unit of the Alfort School of Veterinary Medicine; so far the dog had not received any treatment. The main clinical signs were lethargy, dyspnoea and pale mucous membranes. Blood was collected on EDTA for examination. Blood smears prepared straight after blood collection and observed microscopically revealed the presence of parasites identified as *Trypanosoma*. The dog received an intra-muscular (IM) injection of pentamidine (Pentacarinat^®^) 4mg/kg on December 7th and 8th. After he received pentamidine, the dog showed some improvement, before becoming hypoglycaemic (blood glucose: 0.66 g/L; normal from 0.75 to 1.2 g/L) and hypotensive (systolic pressure = 80 mm Hg; normal from 110 to 169 mm Hg) on December 9th, it died, four days after admission.

Blood collected on admission was sent to the World Animal Health Organisation (WAHO) reference laboratory for African trypanosomes for parasite identification by microscopic and molecular analysis. DNA was extracted using 5 % chelex 100 chelating resin (Walsh *et al.*, 1991) and PCR conducted in 50 µl PCR mixture as previously described, with several taxon-specific primer sets for: *T. (Trypanozoon)*, *T. vivax*, *T. simiae*, *T. congolense* savannah-type, *T. congolense* forest-type, *T. congolense* Kilifi-type, *T. simiae* Tsavo-type, *T. godfreyi*, and Pan-trypanosome primers (TRYP1) (Masiga *et al.*, 1992; Majiwa *et al.*, 1993; Masiga *et al.*, 1996; Desquesnes *et al.*, 2002).

PCR results were unequivocal, and identified the species and type as *T. congolense* forest-type, as described elsewhere. All the other taxa-specific PCRs were negative, and the Pan-trypanosome primers (Desquesnes *et al.*, 2002) produced only one visible product, of the specific size for *T. congolense* forest-type (776bp). It was concluded that this was a mono-specific infection due to *T. congolense* forest-type.

Blood smears were prepared, fixed with methanol, stained with Giemsa and washed under tap water. Once dry, the smears were observed under a microscope, in oil immersion, and photographed with a Canon Power Shot 580^®^ (8.0 megapixels; 28 mm wide angle lens). Relative percentages of the various forms observed were established by counting and grouping parasites on four different blood smears, for a total of 500 parasites. Parasite measurements (magnification × 1,000) were processed directly with a micrometer incorporated in the ocular of the microscope.

For 30 specimens of each morphotype, the following measurements were taken: distance from centre of the nucleus to the posterior extremity (NP), distance from the centre of the nucleus to the anterior extremity (NA), length of the free flagellum (F), distance from the posterior extremity to the centre of the kinetoplast (PK), distance from the centre of the kinetoplast to the centre of the nucleus (NK), diameter of the kinetoplast (K), width of the parasite at its widest point measured on an axis perpendicular to the body (W), length of the nucleus on the axis kinetoplast-nucleus (LN), and width of the nucleus on the axis perpendicular to the kinetoplast-nucleus axis (WN). For all measurements, means (m), standard deviations (S), standard errors (SE = S/√n) and 95 % confidence intervals (CI = 1.96 × SE) were calculated.

The length of the body was defined as L = NP + NA.

The Total Length of the parasite was calculated as TL = NP + NA + F.

The width/length ratio was calculated as WLr = W/L.

Standard indexes were calculated, such as the nuclear index: NI = NP/NA, and the kinetoplastic index: KI = NP/NK.

Light density of the cytoplasm, and coloration aspects of the chromatin in the nucleus were also recorded.

Based on their morphological features, parasites were roughly classified into three main groups, and were counted and classified into one of the three groups on three different slides. The average was calculated to establish the mean frequency of each form. Rosettes occasionally observed were considered as a fourth form. In this paper, to avoid confusion, we use the word “type” only for genetically characterised parasites of the *T. (Nannomonas)* subgenus, and we use the word “form” for the various morphological features of the parasites. Comparisons were made with the description and pictures available in the literature.

## Results

**Fig. 1.**
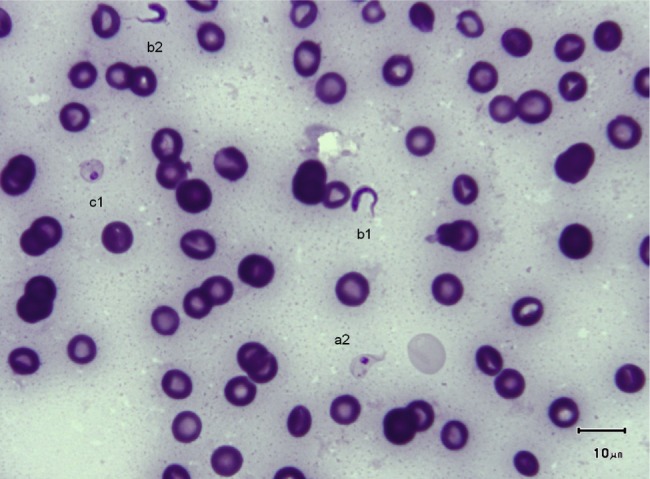
Three main forms of *Trypanosoma congolense* forest-type in a dog: slender forms or *T. (Nannomonas)* isomorph (*congolense*- form) (without free flagellum (b1), with free flagellum (b2)), stumpy form or *T. (Nannomonas)* hyperpachymorph (C-shape: a2) and sphaeroblast-form or *T. (Nannomonas)* sphaeromorph (*montgomeryi*- type: c1). Photo by Marc Desquesnes.

**Fig. 2.**
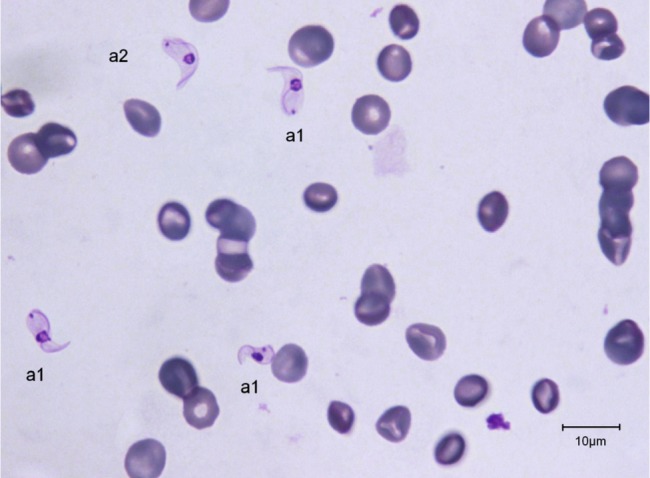
Typical *T. (Nannomonas)* hyperpachymorph with C-shaped (a2) and S-shaped tight flagellum (a1; three specimens). Photo by Bruno Polack.

**Fig. 3.**
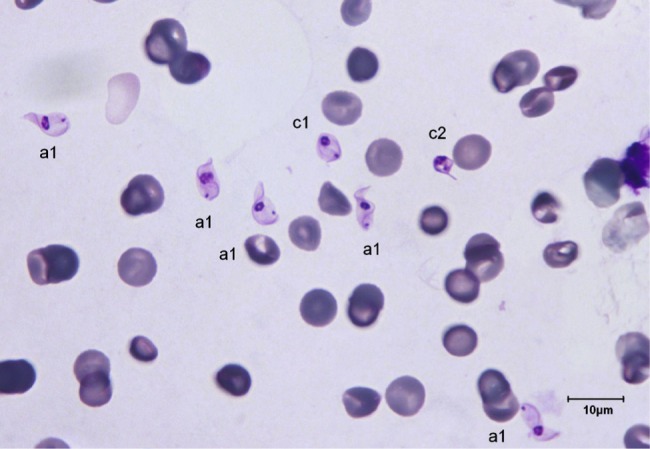
Typical *T. (Nannomonas)* hyperpachymorph (a) (five specimens) and the two types of sphaeroblasts (*T. (Nannomonas)* sphaeromorph) (c). Photo by Bruno Polack. *Montgomeryi*-type sphaeroblast is light in colour and large (c1); *congolense*-type sphaeroblast is densely coloured and small, with a visible free flagellum (c2).

**Fig. 4.**
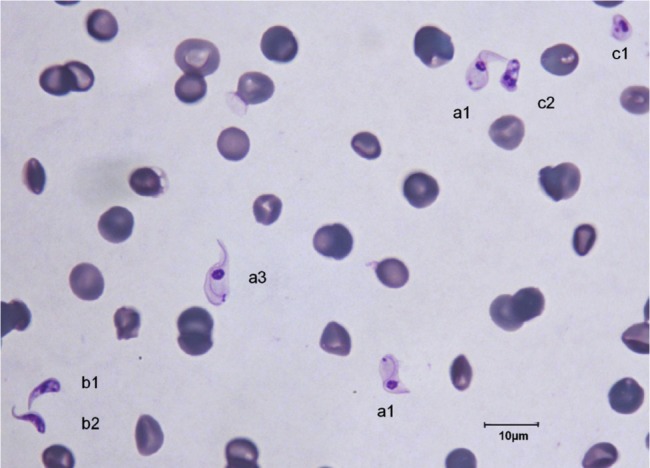
*T. (Nannomonas)* isomorph (b1 & b2), sphaeromorph (c1 & c2) and hyperpachymorph (a1 & a3), the central one (a3) being in division (two kinetoplasts and flagella visible). Photo by Bruno Polack.

**Fig. 5.**
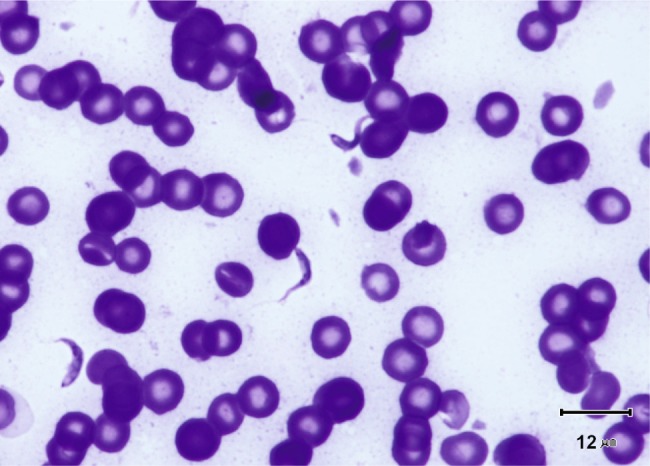
Typical *T. (Nannomonas)* isomorph (*congolense*-forms) without a free flagellum. Photo by Marc Desquesnes.

**Fig. 6.**
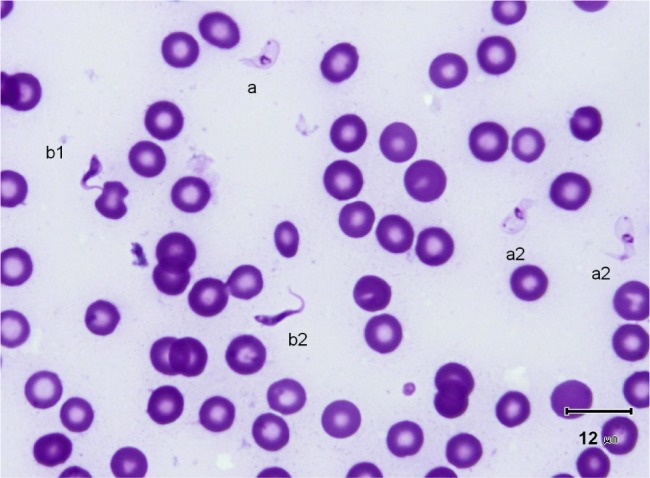
*T. (Nannomonas)* isomorph (*congolense*-forms) without (b1) or with (b2) a free flagellum, and *T. (Nannomonas)* hyperpachymorph (a). Photo by Marc Desquesnes. The upper hyperpachymorph has a C-shaped flagellum (a2), and appears to be almost flat, like a leaf.

**Fig. 7.**
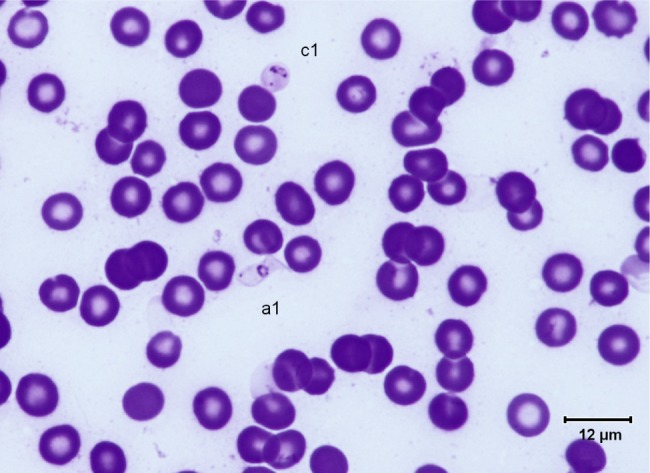
Typical *T. (Nannomonas)* hyperpachymorph (a1) and sphaeromorph (“amastigote”-form) (c1). Photo by Marc Desquesnes.

**Fig. 8.**
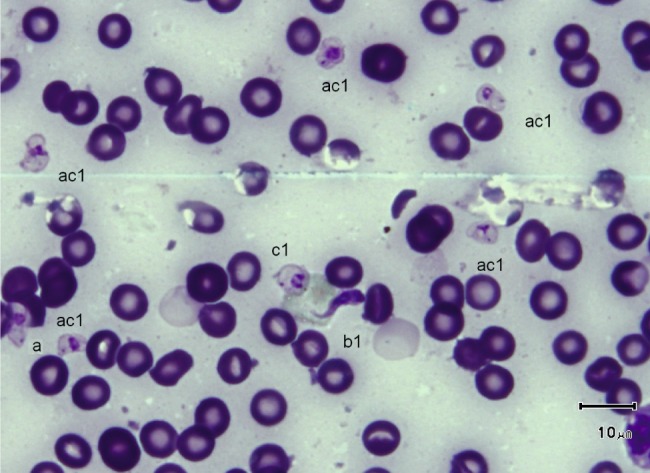
One *T. (Nannomonas)* isomorph (*congolense*-form) (b1) and several forms intermediary between hyperpachymorph and sphaeromorph (ac1). Photo by Marc Desquesnes. Intermediary forms suggest an “S” folding of the parasite on itself like a collapsible tent

**Fig. 9.**
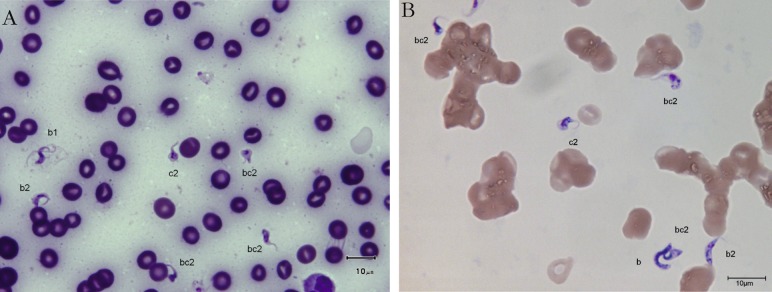
*T. (Nannomonas)* isomorph (b), several forms intermediary between isomorph and sphaeromorph (bc2), and some *T. (Nannomonas)* sphaeromorph (*congolense*-type) (c2). Fig. 9A photo by Marc Desquesnes; Fig. 9B by Bruno Polack.

**Fig. 10.**
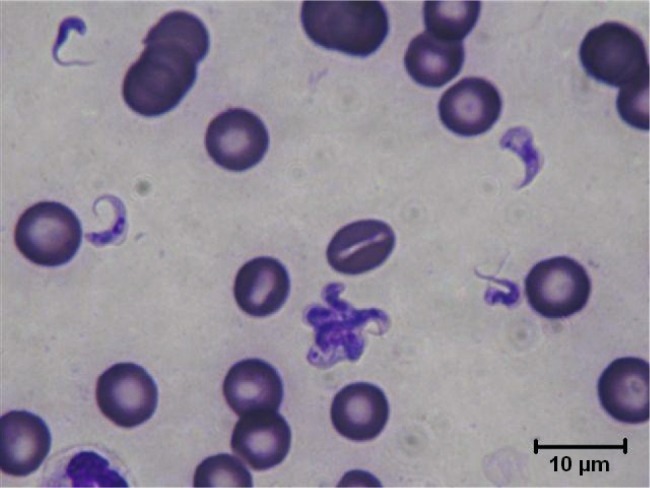
Free forms and Rosette of *T. (Nannomonas)* isomorph. Photo by Sophie Ravel. Several *congolense*-forms: thin and dark with a free flagellum (upper left), medium width and colour, with a large undulating membrane (centre left), broad and light in colour (upper right).

The trypanosomes observed on Giemsa-stained smears were small in size (always under 15 µm in length, and most often under 12 µm) with a small- or medium-sized kinetoplast (0.6–1 µm). In this manuscript, all the figures represent Giemsa-stained blood smears from a dog infected with *Trypanosoma congolense* forest-type; this information is not repeated in the titles and legends of the figures. The parasites exhibited roughly four different forms illustrated and characterised in Figs 1–10 and Table I (at the exception of rosettes): stumpy forms (designed by a letter “a” in the figures), slender forms (b), globular forms (c), and rosettes (d), as described hereafter.

**Table I.** Sizes, frequencies, indexes and other characteristics of the three main morphotypes of *T. (Nannomonas) congolense* forest-type observed on Giemsa stained blood smears.

Morphotype	Hyperpachymorph	Isomorph	Sphaeromorph

Category *	hyper-stumpy form	slender form	globular form
Name	hyper-*montgomeryi*-form	*congolense*-form	sphaerocyte
Percentage observed	48.4 %	30.4 %	20.4 %
NP	4.83 ± 0.3	4.72 ± 0.3	na
NA	4.83 ± 0.4	5.07 ± 0.4	na
NI = NP/NA	1.03 ± 0.1	0.95 ± 0.1	na
F (frequency %) size	absent	(53.3 %) 2.44 ± 0.3	(26.6 %) 1.86 ± 0.3
L = NA + NP	9.67 ± 0.5	9.49 ± 0.8	4.35 ±0.3
Total length TL = NA + NP + F	9.67 ± 0.5	11.09 ± 0.8	na
PK	1.22 ± 0.1	1.02 ± 0.1	1.09 ± 0.1
K (diameter)	0.72 ± 0.1	0.65 ± 0.05	0.72 ± 0.1
KN	4.03 ± 0.3	3.75 ± 0.4	2.25 ± 1.19
KI = NP/KN	1.21 ± 0.04	1.31 ± 0.1	na
W	4.07 ± 0.2	1.97 ± 0.1	4.16 ± 0.4
LN	1.81 ± 0.1	2.32 ± 0.1	1.52 ± 0.1
wN	2.0 ± 0.1	1.25 ± 0.1	2.28 ± 0.2
Cyto light/dark (%)	light	medium or dark	light colour: 70 %; dark colour: 30 %
WLr (min-max)	0.43 ± 0.03 (0.32–0.70)	0.21 ± 0.01 (0.17–0.25)	na
Undulating membrane	S-shaped 87 %; C-shaped 13 %	undulating 100 %	no

For acronyms, see the text; lengths in µm; the 4th morphotype “rosette” (0.8 %) is not presented here (data not applicable – na).

1 – Stumpy forms (frequency: 48.4 %) (Figs 1–3): the mean length of the body is 9.67 ± 0.5 µm, with a width of 4.07 ± 0.2 and up to 5.1 µm, which reflects very stumpy parasites, without a free flagellum (Fig. 2). The cytoplasm is very light in colour, the nucleus is generally circular or oval, and in the latter case seems to be perpendicular to the body (mean length 1.81 µm; mean width 2.0 µm) (Fig. 3), with peripheral clumps of chromatin, or the presence of 2–4 marginal concentrations of chromatin and light colour in the centre of the nucleus. The kinetoplast is small (0.72 ± 0.1 µm), marginal or sub-lateral and sub-terminal (PK = 1.22 ± 0.1 µm) as far as may be estimated, since the posterior extremity is sometimes so large that it appears to be round, and thus the position of the kinetoplast is difficult to locate. The mean KI is 1.21 ± 0.04. Only the nucleus, the flagellum and the kinetoplast are stained; the cytoplasm seems to be empty. There are no convolutions to the flagellum; its shape is most often (87 %) like an “S” which may cross the nucleus or touch its border (Figs 2, 3); the rest of the time (13 %), it is “C” shaped, but in both cases the body of the parasite seems to be tightened by the flagellum. Thus, the “undulating” membrane cannot really be distinguished from the body, which usually looks like a leaf delimited by the flagellum. The position of the nucleus is generally central (NI = 1.03 ± 0.1). This form is similar to the *montgomeryi*form rarely described in the past (Gillain, 1937), but it is not identical, due to the absence of convolutions in the “undulating membrane” and a greater width; the mean width/length ratio (WLr) was 0.43 ± 0.03 (minimum 0.32; maximum 0.70), always > 0.30, and reaching as high as 0.55 or more in some cases.

2 – Slender forms (frequency 30.4 %) (Figs 1, 4, 5): mean body length (without the free flagellum, when present) is almost identical to that of the stumpy form, at 9.44 ± 0.8 µm, although it is variable, ranging from 6 to 12.6 µm. The anterior extremity tapers gradually, which may give the incorrect impression of a short free flagellum (Fig. 5), however, in 53.6 % of the specimens, a real free flagellum is present (Fig. 6). Including the free flagellum, the total length (TL) of the parasite is 11.09 ± 0.8 µm. The parasite is usually narrow, with a mean width of 1.97 ± 0.1 µm, although it can attain 2.4 µm in some specimens; the WLr is 0.21 ± 0.01 (minimum 0.17; maximum 0.27, always < 0.30).The cytoplasm and the nucleus are deeply stained and most often homogeneous in colour; the nucleus is almost central (NI = 0.95 ± 0.1), with the long axis parallel to the body (mean length 2.32 µm; mean width 1.25 µm), however in some instances its borders are not clearly visible due to the dense coloration of the cytoplasm (Fig. 1). The kinetoplast is small (0.65 ± 0.05 µm), clearly sub-terminal (PK = 1.02 ± 0.1 µm, KI = 1.31 ± 0.1) and marginal or sub-marginal, as classically described for *T. congolense*. The flagellum is convoluted, and the undulating membrane is most often conspicuous but weakly developed (Fig. 6). These forms are close to the *congolense*-form (isomorph) previously described (Gillain, 1937).

3 – Globular forms (frequency: 20.4 %) (Figs 1, 3, 7- 9): roughly spherical, with a mean diameter of 4.35 ± 0.3 µm, or oval. They have a small kinetoplast (0.72 ± 0.1 µm) situated more than 1.09 ± 0.1 µm from the extremity (subterminal). 30 % of them are densely stained, have an irregular shape and a discernible flagellum, most of the time partly free, giving them the aspect of sphaeromastigotes (Fig. 9); it may be speculated that these forms derive from the densely-stained slender *congolense*-form parasites. 70 % of them are light in colour and have no free flagellum (Fig. 1); it may be speculated that these forms derive from the lightly stained stumpy parasites; the nucleus is circular in 40 % of the cases, while in 60 % of the cases it is oval and perpendicular to the kinetoplast-nucleus axis (mean length 1.52 ± 0.1 µm; mean width 2.28 ± 0.2 µm) (Fig. 7). These lightly stained forms may have a discernible flagellum, but it is never really free (Fig. 8). All these forms may be called “sphaeromastigotelike”. Some parasites have no visible flagellum, giving the look of amastigotes (Fig. 7), although it appears that the flagellum is in fact present at the periphery of these circular cells (Fig. 1). These forms are close to the “latent-body forms” (or sphaeromorph) previously described (Gillain, 1937).

**Fig. 11.**
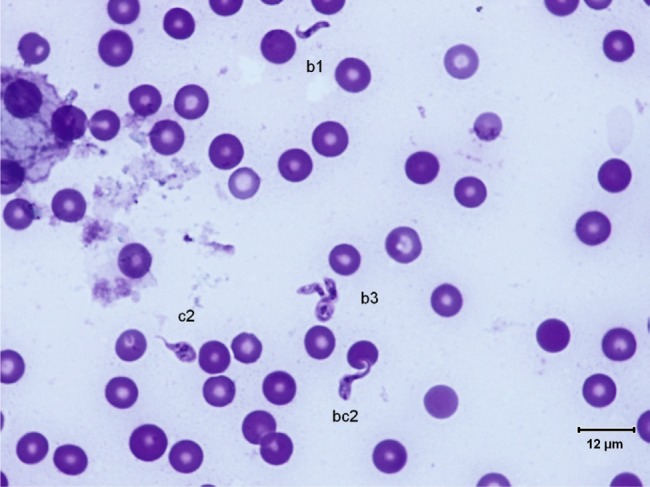
*T. (Nannomonas)* isomorph typical (b1), in abnormal division (b3), and intermediary forms (bc2) toward the *congolense*- sphaeromorph with a free flagellum (c2). Photo by Marc Desquesnes.

**Fig. 12.**
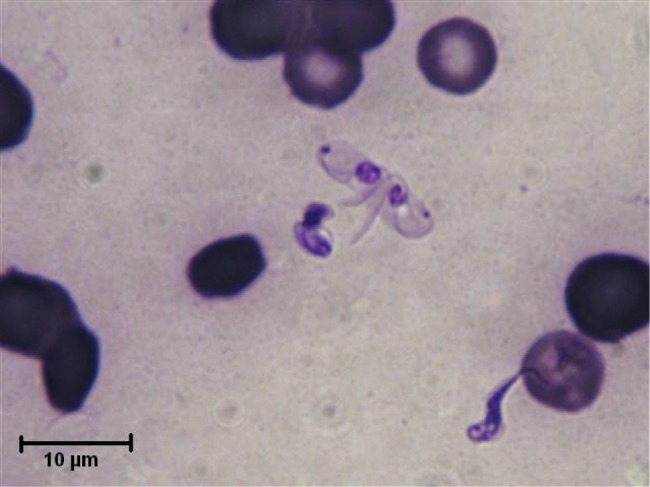
Twin *T. (Nannomonas)* hyperpachymorph and one isomorph. Photo by Sophie Ravel.

4 – Rosettes (frequency: 0.8 %) (Figs 10–12): in some cases parasites do not separate properly during cell division; rosettes have been observed (Fig. 10), as well as abnormal forms (Fig. 11) and twin parasites (Fig. 12). Only twin trypanosomes were observed in stumpy-form parasites, while twins and rosettes were observed in the densely-stained slender *congolense*forms.

**Fig. 13.**
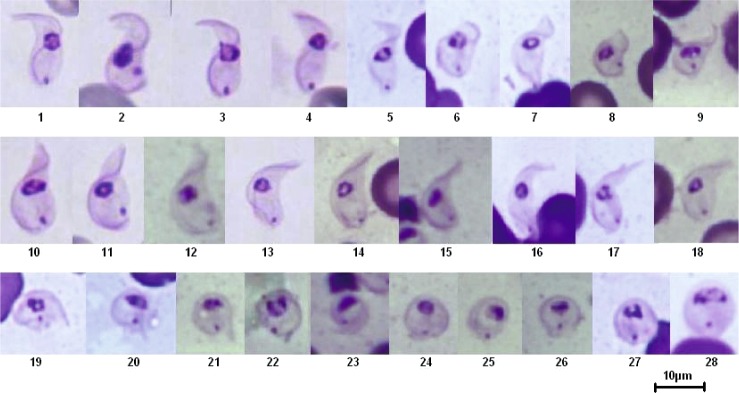
Tentative serial “Cshape” transformation of the *T. (Nannomonas)* hyperpachymorph into sphaeromorph (unfolding and “C-folding” model). Photo by Marc Desquesnes. In this series, the “S-shape” of the flagellum seems to unfold from the posterior end of the kinetoplast toward the anterior end (slides 1–12), to attain a “C” shape (snapshots 13–17) in which the cytoplasm is leaf-shaped (snapshot 16–17), before refolding again like a spring while the anterior end seems to enfold the posterior (snapshots 18–20) to look like a baby fish (snapshots 21–24) before finishing like an egg (snapshots 25–28).

**Fig. 14.**
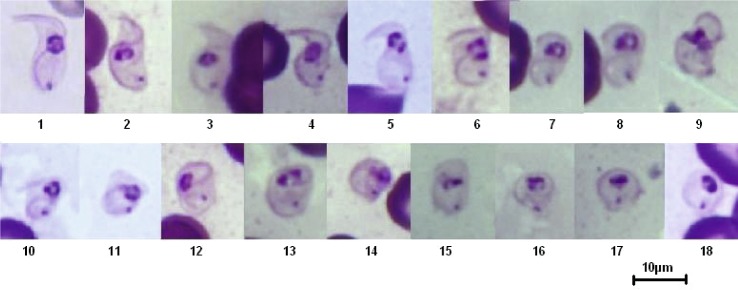
Tentative serial “Sshape” transformation of the *T. (Nannomonas)* hyperpachymorph to the sphaeromorph (“tent folding” model). Photo by Marc Desquesnes. In this series, the “S” shape of the flagellum seems to fold on itself like a spring (snapshots 1- 6), so the anterior and posterior ends move closer to each other like a collapsible tent (snapshots 7–12). The cytoplasm ends up in a sphere (snapshots 13–18).

Intermediary forms, situated between the typical stumpy form and the typical sphaeromastigote have been observed, and serially classified in an attempt to describe the transformation from hyperpachymorph to sphaeromorph (Figs 13, 14). Two different developmental paths may be hypothesised, depending on the transformation of the flagellum shape: in one case (Fig. 13), the initial “S” shape seems to untwist (Fig. 13, snapshots 1–12) and proceed toward a “C” shape (Fig. 13, snapshot 13–15), to end in a circle or a “key holder”, since the flagellum is longer than the perimeter of the sphaeroblast (Fig. 13, snapshots 19–28); in the other case (Fig. 14), the “S” shape seems to twist more (Fig. 14, snapshots 1–6) and to fold on itself like a collapsible tent, making a short spiral flattening on itself (Fig. 14, snapshots 7–15), ending in a sphere or a disk (Fig. 14, snapshots 16–18).

## Discussion

### Comparison to the Morphotypes Reported in *T. (Nannomonas)*

Before molecular tools appear, *T. (Nannomonas)* was thought to contain two species: *T. simiae* and *T. congolense*, presenting five morphological forms in various proportions (Gillain 1937): *congolense*-form, *simiae*-form, *rodhaini*-form, *montgomeryi*- form, and sphaerocyte-form (latent bodies, so-called “amastigote” or “sphaeromastigote” forms).

In this study, the morphological features of the parasite are undoubtedly those of the sub-genus *T. (Nannomonas)*, more specifically the *congolense*-forms which have been observed in a previous similar case of a dog returning from Senegal (Dorso *et al.*, 2010). However, the very stumpy form of half of the parasites is not expected from *T. (Nannomonas)* in a mammalian host. Even in their stumpy forms, the WLr of the *T. congolense*, *T. simiae* or *T. suis* described so far were always < 0.4. Moreover, their flagellum and undulating membrane presented several “convolutions” (see Mulligan 1970, p 57, and Peel & Chardome 1954, Figs I and V–VIII), whether the one described here is of a regular “S” shape (Fig. 2, upper parasite on the right) or, occasionally, “C” shaped without undulations (Fig. 2, upper parasite on the left).

A peculiar very large form of *T. (Nannomonas)* has been described by several workers in the past as *Trypanosoma montgomeryi*. Such forms were mentioned and discussed by several groups of workers (Laveran & Mesnil, 1912; Mulligan, 1970; Hoare, 1972; Toure *et al.*, 1976); they all agree to consider *montgomeryi*- forms as an atypical form which may be observed in pigs, cattle and dogs in *T. congolense*, *T. simiae* or *T. suis* infections; however, in most cases this peculiar form occurs in low percentages, often below 2.5 %. This was obviously not the case here, since the rate of this form was very high (> 48 %).

### Exceptional Morphotype

In the descriptions and illustrations of GSBS exhibiting predominant *montgomeryi*-form parasites there are no parasites of extreme width, as we observed in this dog. The *montgomeryi*-forms described by Montgomery and Kinghorn in a Rhodesian cattle are 11–19 µm long and 3.00–3.75 µm wide, with a WLr < 0.34; those described by Toure had a WLr < 0.32 ; those reported by Hoare and Mulligan were respectively < 0.29 and < 0.36, while those reported by Peel and Chardome were between 0.26 and 0.37 (Laveran & Mesnil, 1912; Peel & Chardome, 1954; Mulligan, 1970; Hoare, 1972; Toure *et al.*, 1976). The widest *T. (Nannomonas)* that was reported in the literature is a drawing of *montgomeryi*form described as a *T. suis* (Wenyon, 1926) quoted by Peel & Chardome (1954) with a WLr of 0.42. Despite the controversy of its attribution to *T. (Nannomonas)* versus Picnomonas, this is the only specimen that could “compete” with the high WLr recorded in our observations. However, membrane convolutions are visible in these parasites (see Peel & Chardome 1954, Fig. VI), while this is not the case in our observations, since the flagellum always seems to be tight and tightening the cytoplasmic membrane (Fig. 2).

In the present case, we observe around 50 % of the hyperpachymorph with a high rate (> 30 %) presenting a WLr > 0.49, and as high as 0.55 or more, to the point where the shape does not allow establishing this ratio due to the spherical aspect of the body. Moreover, the flagellum and undulating membrane have no convolutions, but have an “S” or “C“ shape which seems to be rigid, tightening the cytoplasmic membrane (Fig. 2). This is the first report in which the predominant form of the parasite has a mean WLr of 0.43; we suggest naming it *T. (Nannomonas)* hyperpachymorph, as further justified.

### Meaning of this Form?

In the past, when *montgomeryi*-forms were observed, it has always been at low rates, at the exception of case observed by Montgomery, Kinghorn and Gillain (quoted by Gillain, 1937), in a dead pig infected with *T. simiae*; in this case only *montgomeryi*-form and latent bodies were observed. Such forms seem to appear on two occasions, either in pre-agonic period or recent post-mortem (thus when the infection is fatal to the host), or, when the host controls the infection and kills the parasites (Gillain, 1937); both cases being initial phases of parasite destruction. This was not the case here; the animal was obviously not in preagonic period since it survived four days more, and the parasites as well were not dying, since they ended by killing the host. In contrast, in our observation, even the very stumpy forms are not in the process of being destroyed, since we can observe several specimens multiplying (Fig. 4). Nevertheless, these authors suggest that *montgomeryi*-forms are an intermediate form in the development of the latent body. Our serial observations also suggest such transformation. In the present study, the death of the dog was not imminent (it died four days later), and a very high proportion of *T. (Nannomonas)* hyperpachymorph were observed (nearly 50 %), together with 30 % slender forms and 20 % latent bodies. Latent bodies seem to be of two types: some derived from *congolense*-form (irregular sphaerocyte shape, densely stained with a free flagellum) (Figs 9, 11) while most seem to derive from the *T. (Nannomonas)* hyperpachymorph (circular, light in colour, without a free flagellum) (Figs 3, 7).

When examining forms intermediary between hyperpachymorph and light-density sphaerocytes, it seems that the cytoplasm is tightened by the flagellum and is developing from the helicoidal shape of the former towards a circular shape in the latter through one of two different paths: unfolding into a “C” shape and refolding into a “key-holder” shape, or folding like a collapsible tent. At the end of this process the sphaerocytes obtained are circular in shape, delimited by the flagellum (Fig. 1). In the series of pictures tentatively assembled in Figures 13 and 14, transformation from hyperpachymorph to sphaeromorph, even though the flagellum is no longer visible in the latter stage (because it is at the periphery of the sphaeromorph), it has not been eliminated. If this hypothesis is correct, the term “amastigote” would not be appropriate for these forms, since they still have a flagellum. The absence of parasite isolation put an end to the possibility of confirming the hypotheses suggested by these observations.

Such apparently “amastigote-forms” and “sphaeromastigote- forms” observed here have been previously described both in *T. (Nannomonas)* and *T. (Trypanozoon)* parasites (Erdmann, 1915; Gillain, 1937); as previously stated, they are sometime considered deleterious shapes (apparently not the case here), or latent forms, so-called “latent bodies”. Direct transformation into round bodies has even been observed by some authors under the microscope, within a period of 30 minutes (Fantham, 1911). The fact remains that whether or not the “latent body” is a dead end has always been controversial (Gillain, 1937; Gallais *et al*., 1953). In our observations, the evolution from hyperpachymorph to sphaeromorph does not appear to be deleterious, since it is regularly spherical and similar in several pictures observed (Figs 13, 14), while that of *congolense*-forms to sphaerocytes appears to be deleterious, since it is irregularly spherical and appears to be accidental, with hazardous morphological changes (Figs 3, 4, 9). However, interpretation of pictures in that way would also, by definition, remain questionable, so the authors are unable to reach a conclusion on this point.

## Conclusion

To the best of our knowledge, in the present report of a monospecific *T. congolense* foresttype infection in a dog, a high rate (48 %) of stumpy-form parasite is broader than any other *T. (Nannomonas)* yet described (including *montgomeryi*). We propose adding this atypical form to the morphotypes admitted for *T. congolense* sensus lato.

As suggested by Toure (1976), to complete his proposal and avoid reference to species names, parasitic stages or author’s names, the following vocabulary could be adopted to describe the trypanosomes of the subgenus *T. (Nannomonas)*:

- *T. (Nannomonas)* hyperleptomorph: very long and slender, with a free flagellum (ex *rodhaini*-form) (not observed in this study);
- *T. (Nannomonas)* leptomorph: long and slender, with a free flagellum (ex *simiae*-form) (not observed in this study);
- *T. (Nannomonas)* isomorph: slender, generally without a free flagellum (ex *congolense*-form) (observed as “slender forms” with or without free flagellum in this study);
- *T. (Nannomonas)* pachymorph: stumpy, short and stout, 0.25 < WLr < 0.34; without a free flagellum (occasionally described with a free flagellum in *T. suis*) (ex *montgomeryi*-form) (incidentally observed in this study but not mentioned);
- *T. (Nannomonas)* hyperpachymorph: very short and very stout, 0.35 < WLr < 0.7, without a free flagellum, cytoplasm light in colour (hyper-*montgomeryi*-form), tightened by a prominent “S” (/”C”) shaped flagellum, without convolution of the undulating membrane (described as “stumpy forms” in this study);
- *T. (Nannomonas)* sphaeromorph: sphaeromastigotes, WLr > 0.8, previously described as “latent bodies” or “amastigotes” (observed as globular forms in this study).

On top of this list, rosettes of various morphotypes might be observed, like in other sub-genera.
